# Myeloid Sirtuin 2 Expression Does Not Impact Long-Term *Mycobacterium tuberculosis* Control

**DOI:** 10.1371/journal.pone.0131904

**Published:** 2015-07-02

**Authors:** Filipa Cardoso, Flávia Castro, Lúcia Moreira-Teixeira, Jeremy Sousa, Egídio Torrado, Ricardo Silvestre, António Gil Castro, Margarida Saraiva, Teresa F. Pais

**Affiliations:** 1 Life and Health Sciences Research Institute (ICVS), School of Health Sciences, University of Minho, Braga, Portugal; 2 ICVS/3B’s—PT Government Associate Laboratory, Braga/Guimarães, Portugal; 3 Instituto de Medicina Molecular, Av. Prof. Egas Moniz, Lisbon, Portugal; Fundació Institut d’Investigació en Ciències de la Salut Germans Trias i Pujol. Universitat Autònoma de Barcelona. CIBERES, SPAIN

## Abstract

Sirtuins (Sirts) regulate several cellular mechanisms through deacetylation of several transcription factors and enzymes. Recently, Sirt2 was shown to prevent the development of inflammatory processes and its expression favors acute *Listeria monocytogenes* infection. The impact of this molecule in the context of chronic infections remains unknown. We found that specific Sirt2 deletion in the myeloid lineage transiently increased *Mycobacterium tuberculosis* load in the lungs and liver of conditional mice. Sirt2 did not affect long-term infection since no significant differences were observed in the bacterial burden at days 60 and 120 post-infection. The initial increase in *M*. *tuberculosis* growth was not due to differences in inflammatory cell infiltrates in the lung, myeloid or CD4^+^ T cells. The transcription levels of IFN-γ, IL-17, TNF, IL-6 and NOS2 were also not affected in the lungs by Sirt2-myeloid specific deletion. Overall, our results demonstrate that Sirt2 expression has a transitory effect in *M*. *tuberculosis* infection. Thus, modulation of Sirt2 activity *in vivo* is not expected to affect chronic infection with *M*. *tuberculosis*.

## Introduction

Sirtuins (Sirts) are a family of seven NAD^+^-dependent protein deacetylases in mammalians (Sirt1-Sirt7), distributed by different cellular compartments and enabling cells to deal with several stress conditions [1,2,3]. For example, Sirt1, 2 and 6 were shown to target several substrates involved in the cellular stress associated to inflammatory responses, such as the transcription factors NF-κB [4,5,6], AP-1 [7] and Foxp3 [8]. Specifically, Sirt2 shuttles between cytosol and nucleus effectively removing the acetyl group from lysine 310 of p65 subunit of NF-κB [5] and inhibiting the transcription of several inflammatory genes, including *Il6*, *Il1β* and *Nos2*, in activated macrophages [9,10,11,12]. Sirt2 also targets tubulin, inhibiting the microtubule-driven assembly of NLRP3 inflammasome, thus further supporting an anti-inflammatory role for this Sirt [13]. Indeed, Sirt2 suppresses inflammation in arthritis [14], colitis [12], in 12-*O*-tetradecanoylphorbol 13-acetate (TPA)-induced ear edema [11] and in lipopolysaccharide (LPS)-induced brain inflammation [9]. Despite the evidence supporting a role of Sirt2 in regulating inflammation, the participation of this molecule in host-pathogen interactions only recently began to be unveiled. Infection with the intracellular pathogen *Listeria monocytogenes* induces translocation of Sirt2 from the cytosol to the nucleus where it deacetylates histone H3 on lysine 18, thus inducing subsequent gene repression [15]. This effect is mediated by the listerial virulence factor protein InlB, which is essential for in vivo infection by *L*. *monocytogenes* [15]. Although absence of Sirt2 clearly impaired *L*. *monocytogenes* infection [15], little is known on its impact in infections by other intracellular pathogens, such as *Mycobacterium tuberculosis*, the causative agent of tuberculosis.

A growing body of evidence highlights that the balance between the host immune response and *M*. *tuberculosis* factors is critical to the outcome of infection, with perturbations in the inflammatory profile potentially leading to a faster bacterial replication, accompanied by disease [16,17,18,19,20]. Because macrophages are within the first cells becoming into contact with *M*. *tuberculosis* during infection, the initial events dictating the innate immune response by myeloid cells not only impact the local and immediate inflammatory immune response, but also potentially shape the intensity and quality of the subsequent acquired immune response. Taking into consideration the role of Sirt2 in modulating the inflammatory response and the importance of this response in the context of *M*. *tuberculosis* infection, here we investigated whether the expression of Sirt2 in myeloid cells regulates the course and outcome of *M*. *tuberculosis* infection. Although Sirt2 initially impacted control of bacilli proliferation, this effect was attenuated at long-term. Overall, our results show that myeloid expression of Sirt2 is not critical in *M*. *tuberculosis* infection.

## Materials and Methods

### Ethics Statement

All animal experiments were performed in strict accordance with recommendations of the European Union Directive 2010/63/EU and previously approved by Portuguese National Authority for Animal Health–*Direção Geral de Alimentação e Veterinária*. Mice were euthanized by CO_2_ inhalation with efforts to minimize suffering.

### Animals

LysM-Cre^+^Sirt2^fl/fl^ mice were obtained by crossing LysM-Cre mice (The Jackson Laboratory) with Sirt2-floxed mice used through an MTA with Johan Auwerx & Kristina Schoonjans Laboratory of Integrative and Systems Physiology, NCEM, Ecole Polytechnique de Lausanne (EPFL), Switzerland. Experimental mice were matched for sex and age and were infected at between 8 and 12 weeks of age.

### Bacteria

*M*. *tuberculosis* H37Rv, originally from the Trudeau Institute Mycobacterial Collection and kindly provided by Dr. A. M. Cooper, was grown in Proskauer Beck medium containing 0.05% Tween 80 to mid-log phase and frozen in 1-mL aliquots at –80°C, as previously described [21].

### Bone marrow derived macrophages

Bone marrow-derived macrophages (BMDM) were differentiated from bone marrow precursors cultured in complete DMEM (cDMEM, containing 10% FBS, 1% sodium pyruvate, 1% HEPES and 1% L-glutamine. all from GIBCO) supplemented with 20% of L929-cell conditioned media (LCCM), as previously described [22]. Briefly, total bone-marrow cells were cultured in microbiological Petri dishes (Sterilin) and kept at 37°C and 5% CO_2_. Cells were fed on day 4 with equal volume of cDMEM containing 20% LCCM. BMDM were recovered on day 7 of the culture, counted, seeded in 24 well-plates and used to infect with *M*. *tuberculosis*. IL-6 production in culture supernatants was measured by ELISA, according to the manufacture’s recommendations (eBiosciences).

### Experimental infection and bacterial load determination

Mice were infected with *M*. *tuberculosis* H37Rv via the aerosol route using an inhalation exposure system (Glas-Col), as previously described [23]. The infection dose was confirmed by determining the number of viable bacteria in the lungs of 5 animals, 3 days after the aerosol infection. The initial infectious dose was Log_10_1.942±0.106; Log_10_2.00±0.030; and Log_10_2.177±0.124, for three independent experiments performed. For bacterial load determination, mice were euthanized and the lungs were aseptically excised, individually homogenized, followed by plating serial dilution of the organ homogenate on nutrient 7H11 agar (BD Biosciences). Colony forming units were counted after 3 weeks of incubation at 37°C.

### Flow Cytometry Analysis

For the analysis of surface markers, 1x10^6^ lung cells were stained with antibodies anti-CD3-PerCPcy5.5 (clone 145-2C11),-CD4-APC-Cy7 (clone GK1.5) and-CD11b-PE (clone M1/70) (all from eBioscience);-CD11c-BV421 (clone N418) and-Ly6G-APC (clone 1A8) (Biolegend) and-Ly6C-PerCPCy5.5 (clone AL-21, Pharmingen). For intracellular staining, 2-3x10^6^ lung cells were restimulated *in vitro* with a mixture of phorbol myristate acetate (PMA; 50ng/mL) and ionomycin calcium salt (500ng/mL), in the presence of brefeldin A (10μg/mL; all from Sigma-Aldrich) for 4 hours at 37°C. After restimulation, cells were fixed and stained for surface CD4 and intracellular IL-17, IFN-γ and TNF as described before [23]. Samples were acquired on a LSRII flow cytometry with Diva Software. All data were analyzed using FlowJo version 10 software. The total number of cells in each gate was calculated using the total number of cells determined by Countess Automated Cell Counter. The gating strategy followed is represented in S1 Fig.

### Quantitative Real Time-PCR analysis

Total RNA from infected lungs was extracted with TRIzol Reagent (Invitrogen) according to the manufacturer’s instructions. cDNA was synthesized and analyzed by real-time PCR as described previously [24]. Target gene mRNA expression was quantified using SYBR green (Fermentas) and specific oligonucleotides for each molecule and normalized to the ubiquitin mRNA levels.

### Histological analysis

Anterior lobe of lungs were recovered from infected mice, fixed with 3.7% phosphate-buffered formalin, embedded in paraffin, sectioned in 3μm thickness sections and stained with hematoxylin and eosin (H&E). Immunofluorescence was performed on formalin-fixed tissue sections as previously described [24]. Briefly, antigens were unmasked and blocked with BSA and Fc-Block, and endogenous biotin was neutralized. Sections were probed with purified rabbit anti-NOS2 (M-19, Santa Cruz Biotechnology) followed by a secondary Alexa Fluor 568 goat anti-rabbit IgG (Invitrogen). Vectashield mounting medium with DAPI (Invitrogen) was used to detect nuclei. Images were obtained with an Olympus BX61 microscope and were recorded with a digital camera (DP70).

### Statistical Analysis

The results are given as means ± standard error of the mean (SEM) of at least five animals per experimental group, as indicated in the figure legends. Differences between groups were analyzed by unpaired Student’s *t* test using Graph Pad Prism 6 software, as indicated in the figure legends. Values were considered significant for p≤0.05.

## Results

### Ablation of myeloid Sirt2 transiently impacts the control of *M*. *tuberculosis*

**Fig 1:**
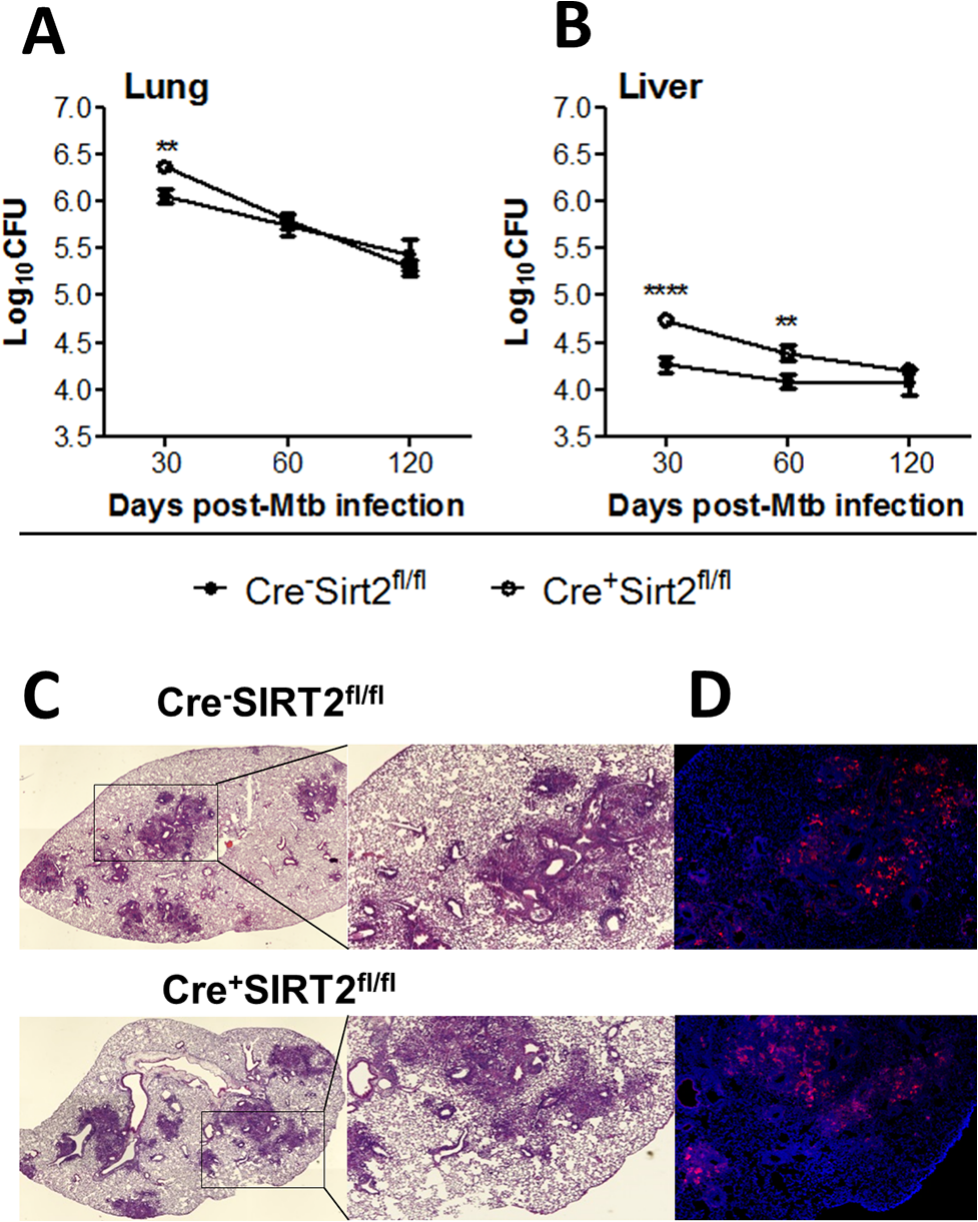
Ablation of myeloid Sirt2 transiently impacts the control of *M*. *tuberculosis*. (A) Lung and (B) liver *M*. *tuberculosis* burdens at days 30, 60 and 120 post-infection of Cre^+^Sirt2^fl/fl^ mice (white circles) or Cre^-^Sirt2^fl/fl^ (black circles). Represented are 3 independent experiments. The initial infectious dose was Log_10_1.942±0.106; Log_10_2.00±0.030; and Log_10_2.177±0.124, for three independent experiments performed. *, p < 0.05; **, p < 0.01; determined by unpaired t-test; (C) Microscopic inflammatory lung lesions of *M*. *tuberculosis*-infected mice stained with hematoxylin-eosin. (D) NOS2 (red) and nuclei (blue) immunofluorescence staining, 30 days post-infection.

Previous studies have demonstrated a role of Sirt2 both in inflammation [9,12,14] and in infection by the intracellular pathogen *L*. *monocytogens* [15]. Since the control of *M*. *tuberculosis* infection is dependent on the activation of macrophages, we questioned whether ablation of Sirt2 in the myeloid lineage would impact the course of *M*. *tuberculosis* infection. We infected control mice (Cre^-^Sirt2^fl/fl^) or myeloid-restricted Sirt2 deficient mice (Cre^+^Sirt2^fl/fl^) with this pathogen via the aerosol route and followed the bacterial burdens over time. At day 30 post-infection, we observed a significant increase of CFUs in the lungs of Cre^+^Sirt2^fl/fl^ mice as compared to controls (difference of log0.305) (Fig 1A). However, by day 60 of infection and onwards bacterial burdens were similar in both groups (Fig 1A). A similar profile of bacterial burden was observed at day 30 post-infection in the liver (difference of log0.523), although the increased susceptibility of Cre^+^Sirt2 ^fl/fl^ mice was prolonged up to day 60 post-infection (difference of log0.305) (Fig 1B). Independently of the organ, the long-term control of *M*. *tuberculosis* was not compromised by the absence of myeloid Sirt2, as on day 120 post-infection bacterial burdens were similar in both groups of mice (Fig 1A and 1B). The difference observed in bacterial burdens on day 30 post-*M*. *tuberculosis* infection was not translated to a different histological pattern of the infected lungs (Fig 1C), nor to distinct NOS2 foci (Fig 1D). These data show that myeloid Sirt2 deficiency has a transient impact in bacterial burden without influencing the pathology caused at the site of infection.

### Absence of Sirt2 in the myeloid lineage does not impact the cellular responses to *M*. *tuberculosis* infection

**Fig 2:**
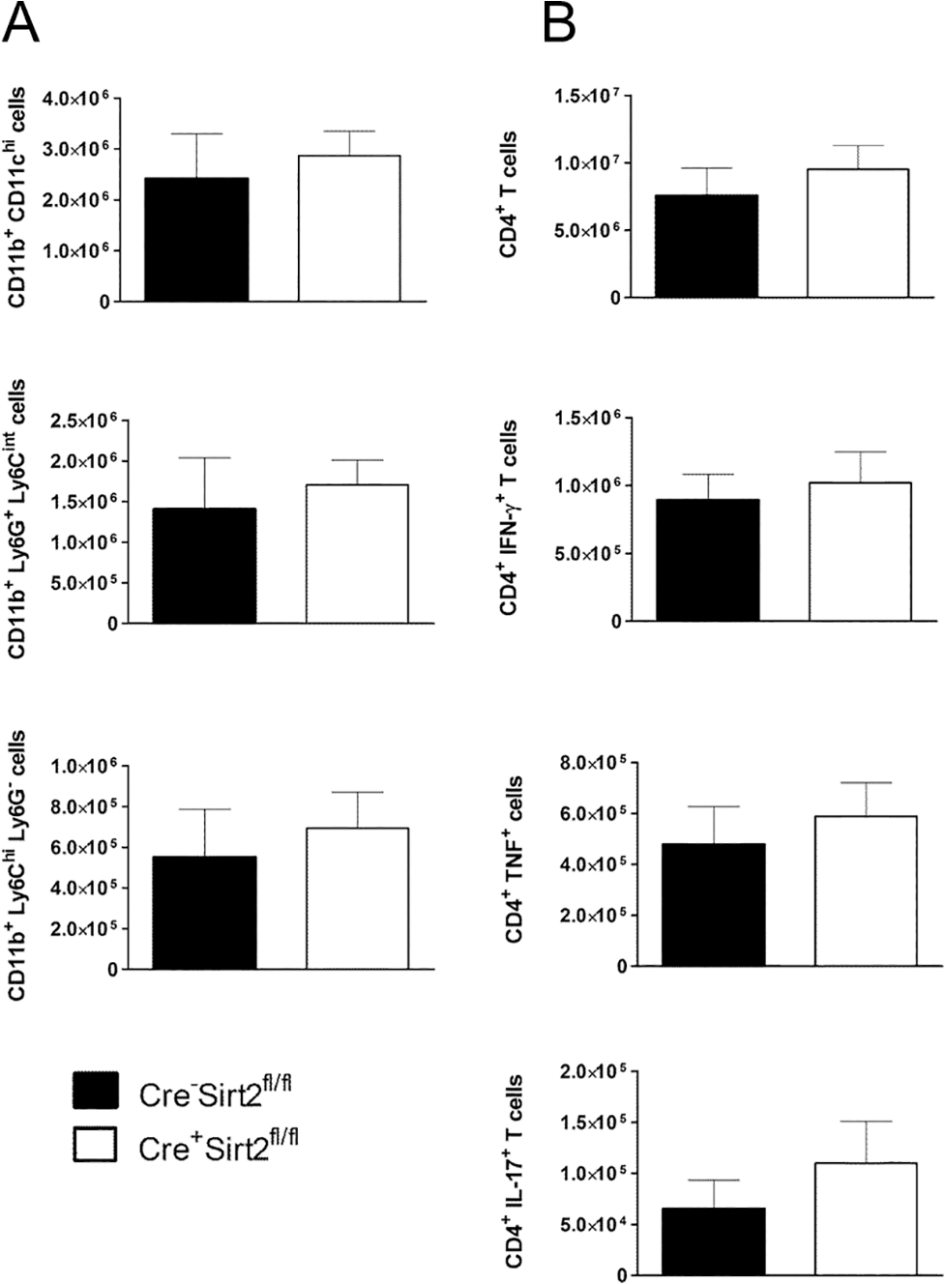
Absence of Sirt2 in myeloid cells does not impact lung cellular responses to *M*. *tuberculosis*. (A) Myeloid cell populations in lung 30 days post-infection were characterized by flow cytometry. (B) Flow cytometry analysis of total CD4+ T cells and IFN-γ, IL-17 and TNF production by CD4+ T cells restimulated with PMA and ionomycin in the presence of brefeldin A. Graphs show the mean ± SEM value of one representative experiment of at least two independent ones (n = 5). The gating strategies and representative plots are in S1 Fig. Significance was determined by the Student’s *t*-test.

Dynamics of cell infiltration to the lungs of infected animals is critical for the establishment of a protective immune response against *M*. *tuberculosis* [25]. Both myeloid cells, resident or recruited, and CD4+ T helper (Th) cells play a role in this process. To address whether the increased susceptibility of Cre^+^Sirt2^fl/fl^ mice on day 30 post-infection was related to a different cellular response at the site of infection, we characterized the myeloid and T cell populations by flow cytometry (S1 Fig). We analyzed different myeloid populations with relevance during the course of infection, including alveolar macrophages (CD11b^+^CD11c^hi^), neutrophils (CD11b^+^Ly6G^+^Ly6C^int^) and inflammatory monocytes (CD11b^+^Ly6C^hi^Ly6G^-^). As shown in Fig 2A, the number of cells in each myeloid population analyzed was similar independently of the expression of Sirt2 in myeloid cells. Similarly, the number of CD4+ T cells in the lung of infected animals was identical in Cre^-^Sirt2^fl/fl^ and Cre^+^Sirt2^fl/fl^, as were the number of CD4+ T cells capable of producing IFN-γ, IL-17 or TNF, as measured by intracellular staining upon *in vitro* restimulation (Fig 2B). Of note, no cytokine-producing cells were detected in non-infected animals (data not shown). Thus, ablation of myeloid Sirt2 neither impacted the lung myeloid cellular populations observed at that time point, nor did it influence IFN-γ-, IL-17- and TNF-mediated protective T cell responses. These data suggest that the increased bacterial burden observed on day 30 post-infection in Cre^+^Sirt2^fl/fl^ animals is not caused by alterations in these cellular mediators.

### Absence of myeloid Sirt2 does not impact the expression of protective molecules during *M*. *tuberculosis* infection

**Fig 3:**
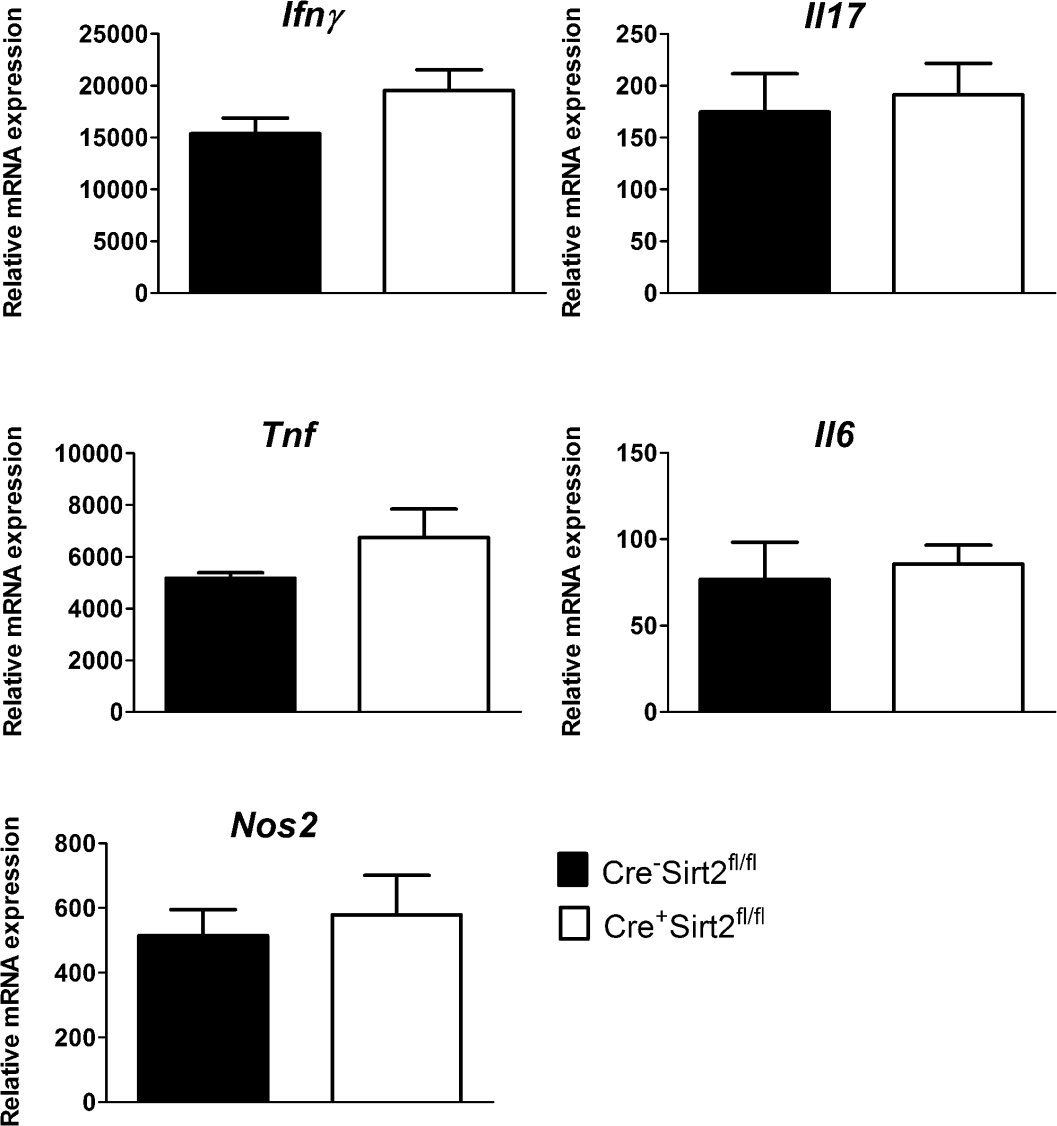
The expression of inflammatory mediators in infected lungs is not altered in the absence of myeloid Sirt2. RNA was extracted from the lung tissue after 30 days of infection and the expression of *Ifn*γ, *Il17*, *Tnf*, *Il6* and *Nos2* was analyzed by real-time PCR and normalized to the expression of ubiquitin. Data shows the mean ± SEM value (n = 5–6) and the significance was determined by the Student’s *t*-test. The data are representative of two independent experiments.

Considering the role of Sirt2 in the transcriptional control of macrophages responses [9,10], we next investigated if the difference observed in bacterial burden was due to distinct expression of protective molecules in the lungs of Cre^-^Sirt2^fl/fl^ and Cre^+^Sirt2^fl/fl^. For that, total RNA of lung extracts was isolated, converted to cDNA and the transcription of *Ifn*γ, *Il17*, *Tnf*, *Il6* and *Nos2* measured by real-time PCR. These molecules have been shown to play important protective roles in the context of *M*. *tuberculosis* infection [25]. Transcription of some these genes has been previously described to be affected by Sirt2 [5,9,11]. In fact, we confirmed that ablation of Sirt2 in BMDM led to increased levels of IL-6 upon infection with *M*. *tuberculosis* (S2 Fig). Consistently with the cytometry (Fig 2B) and the immunofluorescence (Fig 1C) data, the transcription of *Ifn*γ, *Il17*, *Tnf* and *Nos2* was similar in both groups at day 30 post-infection (Fig 3). Additionally, no difference was observed in the transcription of *Il6* (Fig 3). Thus, the overall inflammatory response in the lung of myeloid-restricted Sirt2 deficient mice was similar to that of Sirt2-competent animals, despite a transient increase in the lung bacterial load.

## Discussion

The Sirt family is composed by several evolutionarily conserved protein deacetylases that regulate many cellular processes including metabolism, cell cycle, and longevity [26]. Additionally, a role for Sirt in infection is emerging. Indeed, a function for specific Sirts in infection with Herpesvirus, Hepatitis virus and HIV has been described [27,28,29,30,31] and broad-range antiviral properties have been recently reported to all seven Sirt [32]. As for bacterial infection, myeloid Sirt1 expression was shown to have little influence in Gram-negative toxin-induced shock or Gram-positive bacteremia [33], whereas ablation of Sirt2 profoundly changed the outcome of infection with *L*. *monocytogenes* [15]. In this study, we expanded the research on the role of Sirt2 in infection, by investigating the impact of myeloid Sirt2 expression in *M*. *tuberculosis* infection. Since *M*. *tuberculosis* is an intracellular pathogen whose control is dependent on the activation of macrophages [25], we addressed this issue using myeloid-restricted Sirt2 deficient mice. We found that ablation of Sirt2 in the myeloid lineage had a transient effect in the outcome of infection, with higher bacterial burdens detected on day 30 post infection in the absence of Sirt2. However, this effect was not sustained over time, pointing to a minor role of myeloid Sirt2 in tuberculosis. Our data also show that the increased susceptibility observed was not related to differential myeloid and T cell responses at this time-point. It is possible that either a more subtle immune alteration on the course of infection or a non-immune mechanism, such as metabolic variations commanded by the absence or Sirt2, underlie our observations. In fact, recent studies show that Sirt2 affects the activity of phosphoglycerate mutase (PGAM), a glycolytic enzyme, preventing the Warburg effect in cancer cells [34]. IFNγ-activated macrophages in mycobacterial granulomas undergo same metabolic changes with increased glucose uptake [35], which can be affected in Kuppfer cells by Sirt2 deletion.

It is notable the fact that ablation of Sirt2 deeply affected the outcome of *L*. *monocytogenes* infection [15], whereas in the case of *M*. *tuberculosis* such was not the case. This could be due to the fact that *L*. *monocytogenes* exploits the activity of the host Sirt2 through the expression of the bacterial virulence factor InlB, for which no homologue is found in *M*. *tuberculosis*.

One third of the world’s population is estimated to be infected with *M*. *tuberculosis* and reactivation of tuberculosis is known to occur when the delicate balance established between the host and the pathogen is broken. A great number of clinical trials are reported with either Sirt inhibitors or activators, for example in the context of metabolic or neurodegenerative diseases [36]. Therefore, the clarification of the role of Sirt2 and other Sirts in infection is relevant for future clinical applications.

## Supporting Information

S1 Fig Gating strategy followed for the flow cytometry analysis. (TIF)

S2 Fig Secretion of Il-6 is increased in BMDM in the absence of Sirt2. BMDM were generated from Cre^+^Sirt2^fl/fl^ mice or Cre^-^Sirt2^fl/fl^ and left uninfected (NI) or infected with *M*. *tuberculosis* at a multiplicity of infection of 2 bacteria:1 cell for 24 hours. Supernatants were recovered and the amount of IL-6 quantified by immunoassay. The significance was determined by the Student’s *t*-test. ***p<0.001.
(TIF)
