# Supplementary figures and images for: Myeloid Sirtuin 2 Expression Does Not Impact Long-Term Mycobacterium tuberculosis Control
Source: PLoS One. 2015 Jul 2;10(7):e0131904. doi: 10.1371/journal.pone.0131904 (PMC4489762; doi:10.1371/journal.pone.0131904)

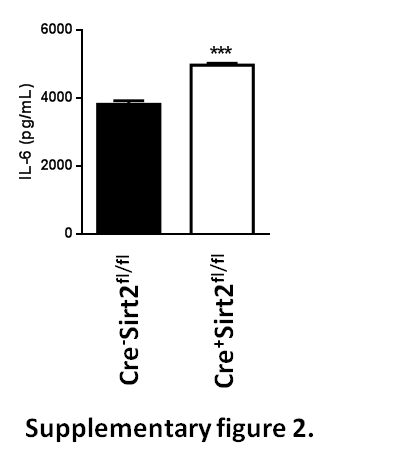

Supplement: S2 Fig — BMDM were generated from Cre+Sirt2fl/fl mice or Cre-Sirt2fl/fl and left uninfected (NI) or infected with M. tuberculosis at a multiplicity of infection of 2 bacteria:1 cell for 24 hours. Supernatants were recovered and the amount of IL-6 quantified by immunoassay. The significance was determined by the Student’s t-test. ***p<0.001. (TIF) [file pone.0131904.s002.tif]
